# Editorial: Nutritional interventions on age-related neurodegenerative diseases

**DOI:** 10.3389/fnagi.2023.1215586

**Published:** 2023-06-13

**Authors:** Tiantian Zhang, Zhigang Liu, Yashi Mi

**Affiliations:** ^1^College of Food Science and Engineering, Ocean University of China, Qingdao, China; ^2^College of Food Science and Engineering, Northwest A&F University, Yangling, China; ^3^Center for Innovation in Brain Science, University of Arizona, Tucson, AZ, United States

**Keywords:** neurodegenerative diseases, nutrition, diet, dietary recommendations, multi-target, sea-land combination

With population aging, tactics to promote healthy aging and counteract the progress of age-related neurodegenerative diseases have become more and more important. A balanced and nutritious diet is vital for keeping fit, especially as age increases. The brain has a high requirement for nutrition, and nutritional imbalance can inhibit its structural and functional integrity, seriously affecting cognitive functioning (Dienel, 2019). Recently, it has been reported that nutritional strategies can decrease the risk of age-related neurodegenerative diseases and play a potentially beneficial role in decelerating the progression of brain diseases and retarding the development of some conditions (Cunnane et al., 2020). A number of investigations have indicated that nutritional interventions could enhance the cognitive ability in sufferers with Alzheimer's disease (AD) (Amini et al., 2020). However, the study connecting the effects of nutritional interventions with age-related neurodegenerative diseases is still in the early stages. It is presently unclear whether dietary constituents affect and regulate brain senescence and neuro regression, especially the molecular mechanisms by which nutritional interventions promote brain health. Developing efficacious nutritional interventions to promote wellness aging is becoming a burgeoning and challenging field.

The prime objective of this Research Topic is to explore the latest important findings related to nutritional interventions for age-related neurodegenerative diseases, and further explore the functional meaning of nutrition on brain health and cognitive ability for the elderly people. The current Research Topic covers 12 articles that provide in-depth insight into the effects of nutritional interventions on age-related neurodegenerative diseases.

A prospective longitudinal study by Sun B. et al. aimed to investigate the association of undernourishment with cognitive performances in the Chinese population using the Chinese Longitudinal Healthy Longevity Survey data from 2011 to 2012, 2014, and 2017 to 2018. There was a significant relationship between the Geriatric Nutritional Risk Index and cognitive performances among the elderly in China. Moreover, individuals with more severe malnutrition have poorer cognitive performance, especially in the oldest illiterate women. The results suggested that clinicians should pay more attention to evaluating the nutritional and cognitive status of the elderly in order to promptly intervene and guard against cognitive dysfunction. In addition, an ancillary MAPT-MRI study by Perus et al. determined the effect of multidisciplinary preventive methods on functional brain connectivity in elderly individuals with cognitive impairment, and the results emphasized the significance of cognitive decline status for preventive interventions.

Currently, dietary recommendations for preventing age-related neurodegenerative diseases are yet not largely accepted in guidelines because of contradictions and limited support. A prospective cohort analysis involving 6,647 men and women aged 55–75 years conducted by Nishi et al. aimed to determine the relation between baseline adherence to three prior dietary patterns, namely the Mediterranean diet (MedDiet), the Dietary Approaches to Stop Hypertension (DASH), and the MedDiet-DASH Intervention for Neurodegenerative Delay (MIND), with 2-year change in cognitive function in elderly individuals who were overweight or obese and at a high risk of cardiovascular disease. In elderly Spanish populations who are overweight or obese and have a higher risk of cardiovascular disease, higher baseline adherence to the MedDiet dietary pattern might be related to better cognitive function, compared to lower adherence within 2 years, while higher adherence to the DASH dietary pattern was unconnected with better cognitive function over a 2-year period. Yang et al. found that the ketogenic diet prevented chronic sleep deprivation-induced AD by decreasing ferroptosis and enhancing the neuronal recovery ability via Sirt1/Nrf2 signaling pathway. The ketogenic diet and caloric restriction will increase ketone bodies, especially β-hydroxybutyrate (BHB). Sun W. et al. found that β-hydroxybutyrate treatment avoided myelin loss, reduced the activation of astrocyte and microglia, and activated the neurotrophin brain-derived neurotrophic factor in the corpus callosum and hippocampus.

Vitamins play a significant role in the maintenance of human physiological functions. A quantitative meta-analysis involving 12 studies (*n* = 1,100) by Hamid et al. tried to determine the connection between vitamin C concentration in plasma and AD while emphasizing the significance and engagement of vitamin C in the etiopathogenesis of AD. Vitamin C deficiency was associated with AD progression, and vitamin C intervention might be a reasonable prevention and treatment method. Notably, clinical research is necessary to illustrate its specific mechanism and role in the pathophysiology and prevention of AD. A case-control study by Cheng et al. involving 360 older people from communities in China evaluated the mediating impact of inflammation on the correlation between vitamin D levels and mild cognitive impairment (MCI). Vitamin D deficiency might enhance the risk of cognitive impairment through a mechanism partially involved in inflammation, thereby vitamin D treatment might improve or delay the cognitive decline caused by inflammation in older people.

Phytochemical ingredients have shown anti-amyloid production, antioxidant, anti-inflammatory and neurotrophic characteristics, which may serve as lead candidates for further prevention of age-related neurodegenerative diseases (Pohl and Kong Thoo Lin, 2018; Yan et al., 2022). Cao et al. detected the levels of β-carboline alkaloids (βCBs), such as harmine, in plasma and tissues of pup rats, aging rats, mice of different physiological states, and healthy volunteers using UPLC-MS/MS and found that the concentration of harmine showed a decreasing trend with advanced age. Zhang et al. found that the food flavoring agent β-caryophyllene could protect neurons and inhibit β-amyloid's neurotoxicity mainly through the JAK2-STAT3-BACE1 pathway. The review “Benefits of dietary polyphenols in Alzheimer's disease” by Wang et al. summarized some of the more promising dietary phytochemicals, especially polyphenols. These substances have been proved to actively regulate some important pathogenesis of AD, such as decreasing β-amyloid plaques and neurofibrillary tangles formation, oxidative stress, neuroinflammation, and synaptic loss. Moreover, this review discussed the latest progress on the potential contribution of gut microbiome in the function of dietary polyphenols.

Seafood is rich in n-3 long-chain polyunsaturated fatty acids, such as docosahexaenoic acid (DHA) and eicosapentaenoic acid (EPA), which play a vital role in brain function (Zhang et al., 2019). Feng et al. found that seafood-derived plasmalogens could protect SH-SY5Y cells against β-amyloid induced toxicity via regulating the transcripts associated with endocytosis, autophagy, apoptosis, neurotransmitter release, and synaptic transmission.

The complex pathogenesis of age-related neurodegenerative diseases results in limited treatment effectiveness, irreversible development of diseases, and significant socio-economic and personal costs (Li et al., 2020). It is necessary to develop a multi-objective diet to prevent the occurrence and development of neurodegenerative diseases. Due to complementary nutritional components, foods from both land and sea contributes to human wellbeing by coordinating resources and nutrient balance. Based on the perspective of the sea-land combination, Wang et al. studied the impacts of Antarctic krill oil (AKO) combined with nobiletin (Nob) and L-theanine (The) on memory loss and cognitive impairment in senescence-accelerated prone 8 mice (SAMP8). Findings showed that AKO exhibited synergistic effects with Nob and The in alleviating recognition memory and spatial memory deficits in SAMP8 mice, respectively. AKO showed synergistic effects with Nob in inhibiting β-amyloid accumulation, neurofibrillary tangles, and apoptosis and neuroinflammation, while the cooperation of AKO and The was involved in synaptic plasticity and anti-neuroinflammation, which indicated that the combination was complex rather than a mechanical addition.

In general, this Research Topic emphasizes the latest advances and innovations in the area of nutritional interventions on age-related neurodegenerative diseases. The Research Topic covers the prevention and treatment of age-related neurodegenerative diseases by the alteration of dietary patterns, macronutrients, micronutrients, and phytochemical compounds. Moreover, the Research Topic covers potential use of combining types of functional ingredients from sea and land toward developing multi-target functional foods. Notably, food is a complex system in which food components can interact, whereby each component can promote or antagonize the effectiveness of other components. In addition, there is inter-individual variability in response to nutritional interventions and their effectiveness for improving cognitive ability. Thus, future research and development directions in this important area should focus on the interaction between food components in alleviating age-related neurodegenerative diseases and personalized nutritional interventions.

## Author contributions

TZ wrote the article. ZL and YM reviewed the article and provided critical feedback. All authors contributed to the article and approved the submitted version.
